# The genome sequence of varicella-zoster virus (*Varicellovirus humanalpha3*) obtained by metagenomics from a patient presenting with an exanthem rash

**DOI:** 10.1128/mra.00248-25

**Published:** 2025-06-10

**Authors:** Sebastian Musundi, Dorcas Okanda, Arnold Lambisia, John Mwita Morobe, Eric Muthanje, Lynette Isabella Ochola-Oyier, Daniel P. Carter, Charles N. Agoti, John Kiiru, George Githinji

**Affiliations:** 1 KEMRI-Wellcome Trust Research Programme, Kilifi, Kenya; 2 UK Public Health Rapid Support Team, London, United Kingdom; 3 Kenya National Public Health Institute, Nairobi, Kenya; 4 Ministry of Health 185929 , Nairobi, Kenya; 5 Department of Biochemistry and Biotechnology, Pwani University 270495, Kilifi, Kenya; Portland State University, Portland, Oregon, USA

**Keywords:** varicella-zoster virus, metagenomics, pox viruses

## Abstract

Here, we report a partial genome sequence of varicella-zoster virus, recovered through metagenomic sequencing from a skin lesion sample collected from a 31-year-old male from Mombasa, Kenya, in August 2024. Phylogenetic analysis placed this isolate in varicella-zoster virus clade 5.

## ANNOUNCEMENT

Varicella-zoster virus (VZV) is a 125 kb linear double-stranded DNA alphaherpesvirus encoding 71 proteins (1). It is the causative agent of chickenpox (varicella) during primary infection and shingles (herpes zoster) upon reactivation after latency in ganglia neurons (2). VZV presents with an exanthem rash, like those observed in other poxviruses, e.g. monkeypox (Mpox) (3, 4). Here, we demonstrate that metagenomic sequence analysis can distinguish these infections.

On 23 August 2024, the KEMRI-Wellcome Trust Research Programme in Kilifi, Kenya, received a skin lesion sample stored in viral transport media (VTMs) (Revital, Kenya) (5) collected from a 31-year-old male resident of Likoni sub-county in Mombasa County for diagnostic confirmation. The patient presented with an exanthem rash, raising the suspicion of an Mpox infection.

Viral DNA was extracted from 140 µL of the VTM sample using the QIAamp viral RNA mini kit and QIAamp Fast DNA Stool Mini Kit (both Qiagen, Germany) with a modified bead beating step (6). DNA was quantified using the Qubit dsDNA High Sensitivity Assay kit on a Qubit 4 fluorometer (Thermo Fisher Scientific, USA). Library preparation employed the Native Barcoding Kit (SQK-NBD114.96) from Oxford Nanopore Technologies (ONT), and sequencing was performed on the ONT platform using an R10.4.1 flow cell. Base calling was conducted using the Dorado (v.7.3.9) software (high-accuracy model), yielding 1.27 million reads, of which 1.07 million reads remained after adapter removal using Porechop (v.0.2.4) (7). For diagnostic purposes, species identification was performed using the Chan Zuckerberg ID nanopore metagenomics bioinformatics pipeline (8). All tools used default settings unless otherwise specified.

To recover the VZV genome, human reads were removed by mapping the sequenced reads against the human reference genome hg38 (GCF_000001405.40) using Minimap2 (v.2.28) (9) and processed using SAMtools (v.1.2) (10). The remaining 193,390 reads were assembled using Flye with the *–meta* option (v.2.8.1) (11), followed by three iterative polishing rounds using Racon (v.1.5.0). The final assembly had an *N*50 of 125,797, and nucleotide BLAST (12) of the 125,797 bp contig matched the human herpesvirus 3 strain isolated from the USA in 2012 (MH709330.1 and MH709332.1), 2013 (MH709360.1 and MH709349.1), and from Germany in 2007 (JN704707.1), with 99.93% identity. Our consensus genome of 124,668 bp with 46% guanine-cytosine content was derived after 8,806 reads mapped to MH709330.1 (99.9% coverage and 34× average depth). Genome annotation was performed in Geneious Prime (v.2024) (Dotmatics, USA) via a homology approach using the closest reference.

**Fig 1:**
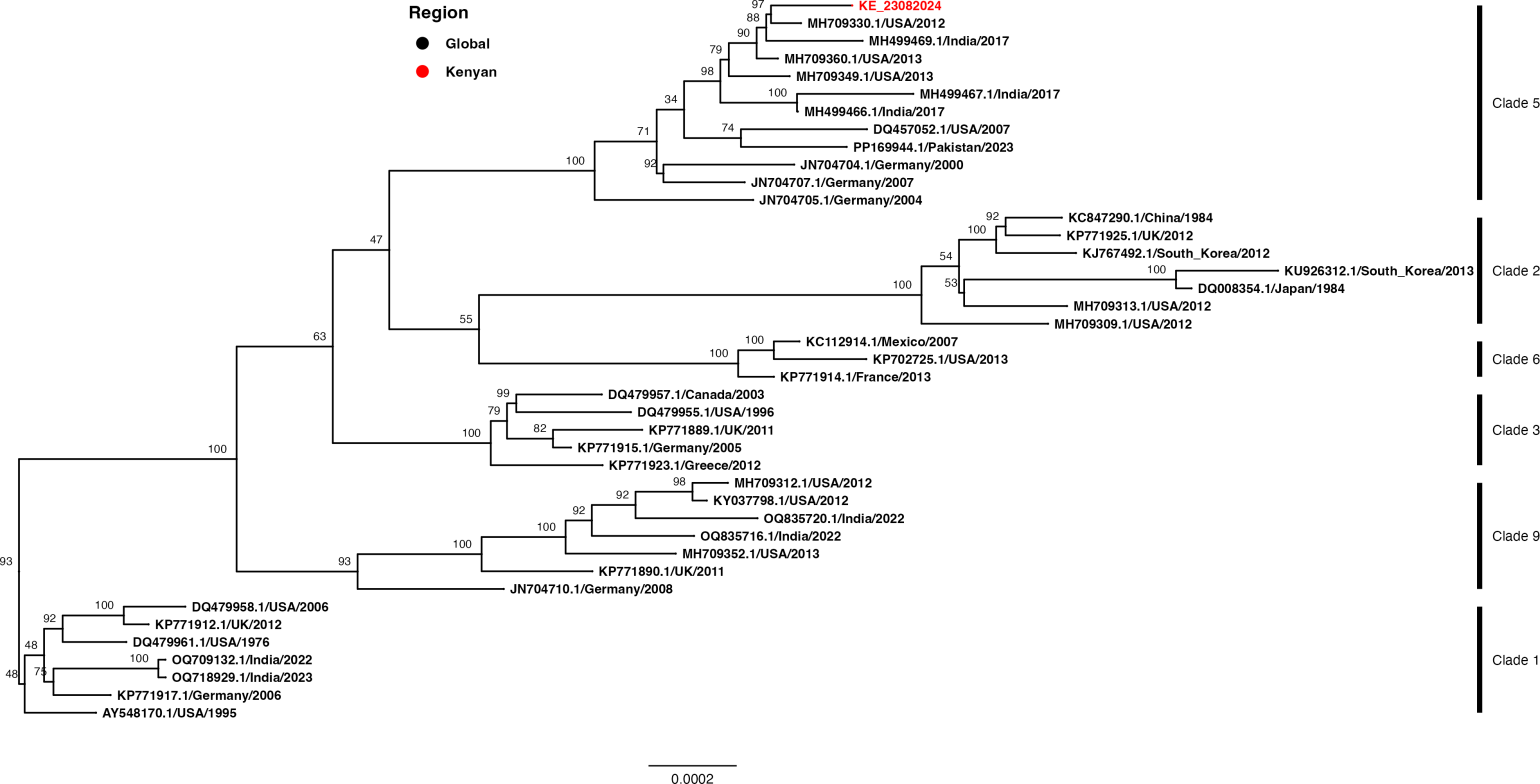
A maximum likelihood tree generated using complete human herpesvirus 3 nucleotide global sequences (*n* = 40) with the Kenyan isolate (KE_23082024) highlighted in red. The tips are colored by geographic origin. Bootstrap values are displayed in the nodes and indicate the level of support for each branch in the phylogenetic tree. The substitution rate is shown in the horizontal bar.

We retrieved VZV genomes (*n* = 40) from GenBank, aligned them using MAFFT (v.7.25) (13), and generated a maximum likelihood tree in IQ-TREE (v.2.3.3) (14) with 1,000 bootstraps using the general time reversible model as selected by ModelFinder (15). The tree was visualized in R (v.4.4.1) using the “treeio” package (16). Based on reported genotyping schemes (17, 18), the Kenyan isolate was classified under clade 5 (Fig. 1).

## Data Availability

Raw sequencing data are available in the Sequence Read Archive (BioProject PRJNA1175889, SRX26453068), and the complete nucleotide sequence is under GenBank accession number PQ505474.
